# A mixed-method pilot study to assess the feasibility of a body–mind intervention in reducing burden and depressive symptoms of informal and semi-formal caregivers of older adults: the DanceCARE research protocol

**DOI:** 10.3389/fpsyg.2026.1770820

**Published:** 2026-05-28

**Authors:** Flavia Galassi, Panos Kassidakis, Marloes van Houten, Rosa-María Rodríguez-Jiménez, Mirian Fernández-Salido, Hanna Poikonen, Marilena Tarousi, Sara Santini

**Affiliations:** 1 Centre for Socio-Economic Research on Aging, INRCA—National Institute of Health and Science on Aging, Ancona, Italy; 2 Aktios Elderly Care Units, Athens, Greece; 3 European Association for Dance Movement Therapy, Munich, Germany; 4 Department of Psychology, Universidad Francisco de Vitoria, Madrid, Spain; 5 Research Institute on Social Welfare Policy, University of Valencia, Valencia, Spain; 6 Wise Motion Community, Helsinki, Finland; 7 Research & Development Department, Computer Solutions S.A., Athens, Greece

**Keywords:** art-based research, body–mind intervention, caregiver burden, dance-movement therapy, depression, long-term care, older adults, wise motion

## Abstract

**Introduction:**

Given the unprecedented increase of long-term care and aging in place demand, informal caregivers are exposed to higher levels of stress (the caregiver’s burden) with negative repercussions on their physical and mental health and additional human and economic costs for the National Health Systems. The DanceCARE method offers an innovative and blended intervention based on a body–mind approach that was co-designed by dance-movement therapists, body–mind experts and aging care researchers to alleviate the burden on informal caregivers of older people. The purpose of the paper is to describe the detailed procedure of the pilot study.

**Method:**

This mixed-methods pilot study aims to measure the impact of the original DanceCARE intervention on the perceived burden and depression levels of 90 informal and semi-formal caregivers living in Greece, Italy and Spain, and the quality of life of older care receivers. Validated psychometric scales, qualitative interviews, and art-based/embodied research methodologies were adopted.

**Results:**

The pilot study will be the first implementation of the DanceCARE intervention, aiming to investigate the feasibility and preliminary effects of the method. This article therefore illustrates the research protocol starting from the main research question and explains the target groups, the pre and post-pilot research phases, the tools that will be used and the resources to employ, including human resources, the time-lines and the methodologies to use uniformly in Italy, Greece, and Spain.

**Discussion:**

The novelty of DanceCARE lies in the body–mind intervention paired with a multi-component assessment methodology that can provide evidence of the efficacy of body–mind interventions that are still under-investigated.

## 1 Introduction

The study is part of the DanceCARE project “Dance Movement Therapy and Conscious Movement as innovative tools in emotional education and support for long-term caregivers” funded by the Erasmus + programme (contract 2023-1-IT02-KA220-ADU-000154702), and aimed to design an original body–mind educational intervention and test it with informal and semi-formal caregivers of older persons with long-term care (LTC) needs in Greece, Italy and Spain.

According to the World Health Organization (WHO) reports, the number of care-dependent people worldwide is increasing every year, and a large percentage (almost 29%) of them are over 60. Co-existing chronic diseases are often associated with the need for health and social care for older adults. Moreover, the European Union’s LTC report (2021) underlines that ensuring sufficient numbers of qualified formal caregivers, i.e., social and healthcare professionals and providing support to informal caregivers, i.e., family members and friends of older people are key challenges in the European aging society. LTC systems are differently structured and developed across Europe, but what all these systems have in common is the predominance of women caregivers in both formal and informal sectors (Wimo et al., 2017).

In many Western countries, particularly those in the Mediterranean region, which are characterized by a family-centered care model (Theobald and Luppi, 2018), in addition to formal and informal services, migrant care workers live with families—namely, private healthcare providers and live-in carers who almost always come from migrant backgrounds and may or may not have a regular employment contract (Stenberg and Hjelm, 2023). They often have not received any training in caring for older adults, but perform daily care and home assistance activities (such as personal hygiene, feeding assistance, and administering medication) that would normally be carried out by professionals in the formal care sector, such as those working in elderly care facilities (Santini et al., 2025). Due to this ambiguity, (migrant) family care assistants can be considered semi-formal caregivers. Live-in migrant family care assistants often experience sleep disorders (Vianello, 2023) financial insecurity, excessive working hours (as many of them live with the elderly person they care for 24 h a day), and social isolation, which compounds the stress associated with their care work and leaves them highly vulnerable (Ulitsa et al., 2025) and lead to poor mental health, e.g., depression and anxiety (Hasan et al., 2021).

By taking both informal and semi-formal caregivers into account, even though they have different motivation to care, and different backgrounds, we take on the challenge of fostering inclusion and building a bridge between these two groups, offering an educational tool that engages them emotionally and encompasses various areas of care. By connecting these two types of caregivers, it is also potentially possible to address the need to match the supply and demand for care.

In general, LT caregivers (both informal and semi-informal) are aging, most of them being middle-aged: a Europe-wide analysis reports that in most countries informal (non-professional) caregivers are between 48 and 53 years old on average (Peña-Longobardo and Oliva-Moreno, 2022). They perform a large part of LTC in most Member States, with important implications for them, the people they care for, and society at large. Both informal and semi-formal care is demanding, and LT informal caregivers are often isolated and at higher risk of psychological distress and depression. Caregiver burden is a specific form of stress (Kim et al., 2012; Lindt et al., 2020) that tends to become chronic the longer the caring situation lasts and manifests itself in the most diverse and subjective forms as sleep and appetite disturbances, depressed mood, and difficulties with concentration and memory, irritability and anxiety, persistent worry, somatization symptoms, such as body ache, and increased susceptibility to illnesses (Bom et al., 2019; Del-Pino-Casado et al., 2019; Greenwood et al., 2018; Mohanty and Niyonsenga, 2019; Pignatiello et al., 2022; Pinquart and Sörensen, 2011). Literature indicates that caregivers who prioritize their own well-being, understand their emotional limits and resources, and avoid isolation by engaging in peer support, are more likely to experience better physical and mental health, leading to less reliance on drugs and healthcare services and improved care for the older person they are looking after (Søvold et al., 2021; Parr and Mielenz, 2023).

Focusing on informal and semi-formal caregivers’ burden and the risk of developing psychological disorders, the scientific community has long discussed the concept of ‘embodied cognition’ and the need to put bodily processes at the center of education, care and mental health (Foglia and Wilson, 2013; Shapiro, 2019). Embodied cognition is a concept that defines the body (sensations, actions and bodily experiences) as a basis for our understanding of the world, the construction of conceptual knowledge, as well as meaning formation (Fincher-Kiefer, 2019; Maes et al., 2014; Varela et al., 1991). Unlike traditional cognitive science, which views the brain as a computer processing symbols, embodied cognition emphasizes a holistic view where the body, environment, perception, and action are all interconnected generating thoughts to emerge. Embodied cognition challenges the idea that cognitive processes are solely about symbol manipulation and abstraction, arguing that many cognitive functions, like perception and understanding, are grounded through the body and arise from direct physical interactions and experiences (Gallese, 2001; Damasio, 2000). This theory has significant implications for understanding learning, language acquisition, social interaction, and even the design of educational tools and methods, where physical actions and experiences can enhance cognitive outcomes. From this perspective, it could be argued that there is no comprehensive intervention if it does not also take the body into account. This concept is slowly spreading and DanceCARE represents a valuable step towards a paradigm shift, responding to the need to reduce the burden of care through body–mind methodologies.

Although there are some encouraging studies demonstrating the potential of body–mind techniques in combating stress and improving mental health and well-being in the adult population (Bräuninger, 2012; Esmail et al., 2020; Rodríguez-Jiménez et al., 2022) there are few studies focusing on informal and semi-formal caregivers of older adults (e.g., Champagne, 2024; Fontanesi and Newman-Bluestein, 2024) and studies supporting a blended online approach for mind–body interventions are also limited and very recent (Blanc et al., 2025; Shim et al., 2024). The DanceCARE protocol was designed to fill this knowledge gap by investigating whether and how a body–mind educational intervention can mitigate the burden and reduce the risk of depression of informal and semi-formal caregivers of older people with LTC needs.

The project aims to provide tools for well-being, care, and health to informal and semi-formal caregivers through Dance Movement Therapy (DMT) and WiseMotion^1^ (WM) methodologies, which have already proven effective in regulating the psycho-emotional stress to which such work inevitably leads. DMT is the psycho-therapeutic use of movement to promote the emotional, cognitive, physical, and social integration of an individual. Grounded in the interconnections of body and mind, DMT uses purposeful movement and creative expression to foster self-awareness, emotional regulation, and resilience (Payne, 1992). Research has shown that DMT is effective in reducing psycho-emotional stress, alleviating symptoms of depression and anxiety, and enhancing overall well-being in both clinical and care-giving populations (Chaiklin and Wengrower, 2015; Pylvänäinen et al., 2015; Karkou et al., 2019). WM is a movement method based on neuroscientific understanding of the bodily and brain functions (Porges, 2009). WM utilizes embodied exercises that combine body awareness, emotional expression, creativity and social interaction to enhance psychological well-being and brain health (Poikonen et al., 2024). Previously, WM has been applied for people with multiple sclerosis disease or dementia, and people recovering from stroke. Both methods emphasize that the first need of a caregiver is to know how to take care of oneself, to have a good awareness of one’s emotions and behavioral reactions, to know how to handle anger, frustration, sadness, stress, and to have a good sense of self.

## 2 Materials and methods

### 2.1 Study design

The main aim of the study is to examine the feasibility of the innovative, blended, and flexible educational program based on DMT and WM methodologies. The second aim is to check if and how the application of key body–mind methodologies contribute to the reduction of burden, depression and isolation of informal and semi-formal caregivers, and at the same time to the increase of the quality of life of older care takers. The study wants to answer the following main research questions “Is the DanceCARE intervention feasible to implement in terms of recruitment, retention, adherence, and acceptability?” and “To what extent and how can the DanceCARE intervention change the perceived burden and depression levels of semi-formal and informal caregivers of older people with long-term care needs?”

Other research questions include whether the intervention also improves the quality of life of the older person receiving care, and whether the blended approach—which involves support through short online videos—is considered helpful and effective by the participants.

To answer these questions, this pilot convergent mixed-method study, chosen in preparation of a future full-scale study, integrates standardized quantitative instruments for measuring perceived levels of burden and depression, with diverse qualitative approaches to capture the complexity of human experience, e.g., interviews, focus-groups and live observations of participants during the body–mind sessions, enriched by art-based and embodied research approach (Hervey, 2000).

These qualitative data will not be limited to verbal accounts; they will also include symbolic and metaphorical expressions emerging from arts-based and embodied practices, which remain grounded within the qualitative paradigm and allow for a process-oriented understanding of participants’ experiences rather than capturing them at a single point in time.

Quantitative and qualitative datasets will be analyzed separately, and the results will then be merged during the interpretative phase. At this stage, aggregate statistical findings will be integrated with qualitative insights, including both quotations derived from interview transcripts and themes identified through the analysis of arts-based and embodied materials. In line with a convergent mixed-methods design, this integration will involve the systematic comparison and triangulation of quantitative and qualitative results, through strategies such as joint displays and interpretative weaving, to identify points of convergence, complementarity, or divergence (Fetters et al., 2013). This integrative process will enable a more nuanced and comprehensive understanding of participants’ experiences with the intervention, combining verbal accounts with symbolic and embodied expressions.

**Figure 1:**
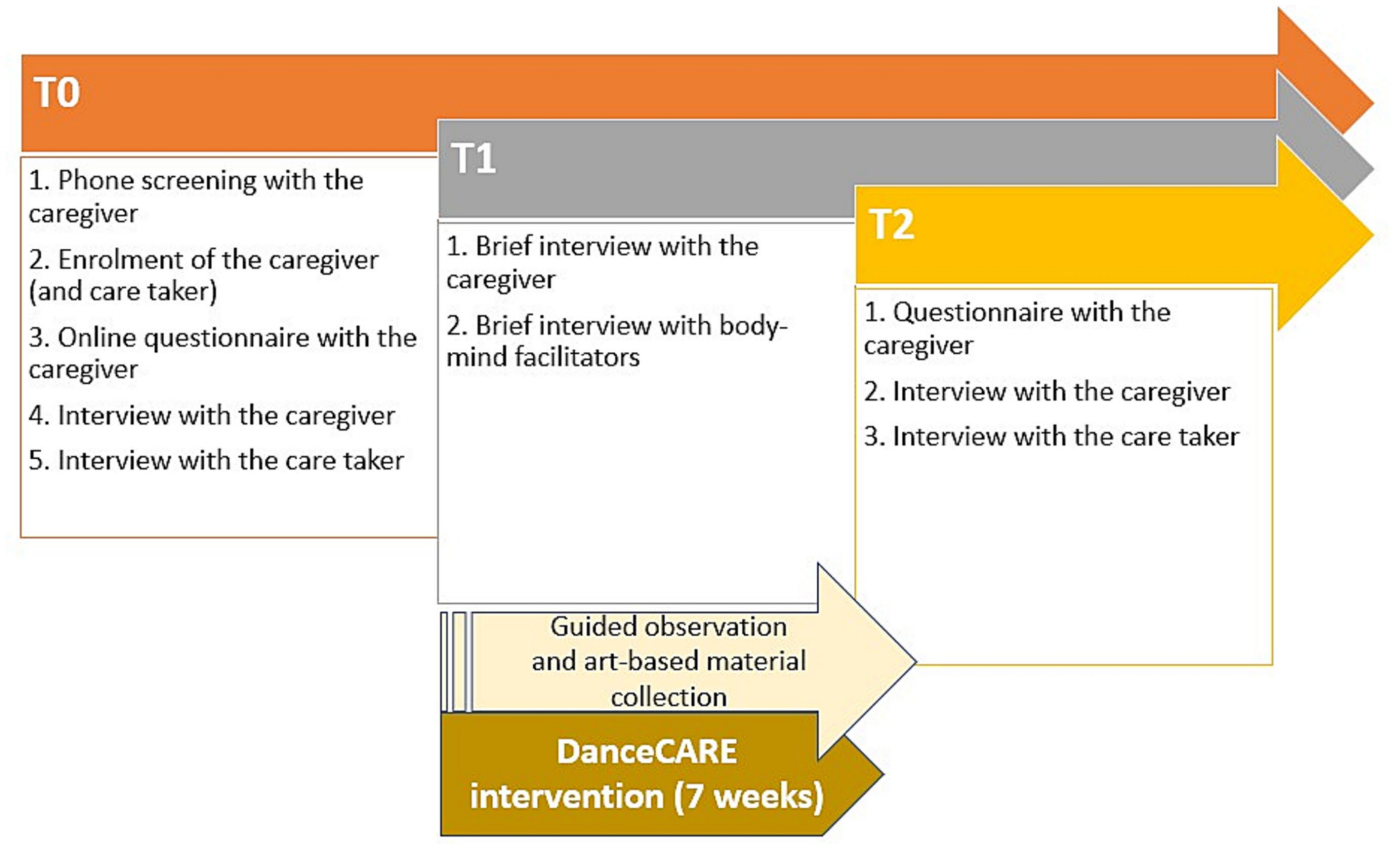
Study design with timeline.

The study foresees three data collection waves: at the baseline (T0) i.e. 3 months before the start of the training; at mid-time, i.e., after the fourth intervention session (T1), and within 2 weeks from the conclusion of the intervention (T2). The three phases of data collection are described in Figure 1.

In the pre-pilot phase, caregivers will be contacted by researchers and screened by phone according to the inclusion criteria (see par 2.2.1.). If the recruited person meets the inclusion criteria, they are included in the sample and they receive the online questionnaires (see Appendix 1) and the informed consent. Contextually, during the phone call, the researcher and the interviewee agree to a face-to-face meeting during which the informed consent is given back signed to the researcher. The latter asks for the possibility of also speaking with the older care recipient, if they are able to answer a very brief interview. Between the screening and the T0 the researcher receives and reads the questionnaire compiled online by the caregiver. At the baseline (T0), the researcher administers the face-to-face interview (see Appendix 2) starting from the answers that the caregiver gave to the questionnaire. When it is possible also the care receiver is interviewed. In the intermediate phase (T1), caregivers and body–mind trainers are briefly interviewed with the aim of understanding whether the activities are liked and effective and whether adjustments need to be made. In the post-intervention phase (T2), the caregivers are asked to answer the questionnaire (containing the same dimensions explored in T0 except for the socio-demographic data) and to participate in a second interview. In this phase, the 15 older adults are interviewed again.

### 2.2 Sample size, recruitment strategy and inclusion criteria

The study aims to reach a total of 30 informal (i.e., relatives, friends, neighbors) and semi-formal caregivers (i.e., migrant family care assistant) and 15 older care receivers per country (total 90 caregivers and 45 older adults), without internal stratification considering the very low number of participants. The study is exploratory and is designed to generate in-depth insights into participants’ experiences rather than to test predefined hypotheses. Accordingly, no formal *a priori* power calculation was conducted, because the sample is intended to provide sufficient informational richness (i.e., “information power”) to address the research aims, rather than statistical representativeness. The sample size was decided upon because the few studies on the effects of dance-based interventions have included samples of this scale (Marmeleira et al., 2009), and others based on the use of multiple artistic mediums such as drawing and music involved approximately 20 subjects (McCarthy et al., 2019; Malyn et al., 2020). Furthermore, given that DanceCARE sample is representative for meaning and not statistically representative (Luborsky and Rubinstein, 1995), a study of the statistical power of the sample is not planned (Jones et al., 2003). Feasibility considerations, including recruitment capacity, available resources, and the intensity of the intervention, further informed the target sample size. To account for potential attrition, a dropout rate of approximately 10–15% has been anticipated, and recruitment will be adjusted accordingly where possible. Participant retention will be monitored throughout the study.

Informal and semi-formal caregivers are recruited using the snowballing method (Valerio et al., 2016), through word of mouth and by spreading leaflets in general practitioners’ ambulatories, geriatric hospitals, territorial health and social services, gyms, pharmacies, voluntary and caregivers’ associations, and through social media, i.e., Facebook and piloting organizations’ websites.

In the three pilot sites in Greece, Italy and Spain, informal caregivers are included if they provide care for an older (65+) family member by choice and/or necessity, without any kind of remuneration for at least 1 year and if they provide at least 12 h of care per week; with a stress self-assessment > of 5 on the stress scale, and if they agree to participate by signing the informed consent. Migrant family care assistants are enrolled if they have been caring for at least 1 year and for at least 20 h per week (Fisher et al., 2021) with a stress self-assessment > of 5 on the stress scale (question no. 8, Caregiver and Supported Person Screening Questionnaire) and if they agree to participate by signing the informed consent. To determine the number of hours, excluding those who live with the person they care for, we relied on recent sector-specific studies that indicate a wide range, with an average that often hovers around 20 h per week for those providing semi-formal care to older people (Verbakel, 2018).

Many informal caregivers are considered “intensive” caregivers when they provide 11 or more hours of care per week. Older adults with long-term care needs are enrolled if they are aged 65 years and over (both men and women); if they have a score on the IADL scale (Lawton and Brody, 1969) between 4 and 6 (indicating an average level of dependency which, however, does not preclude the ability to answer the questions of the questionnaire) and if they agree to participate by signing the informed consent. No other socio-demographic criteria are adopted for selecting participants (see Table 1).

**Table 1:** Participants’ selection criteria.

Type of participants	Inclusion criteria	Exclusion criteria
Informal caregivers	– To provide care for an older family member by choice and/or necessity, without any kind of remuneration	– Receiving payment for caregiving
	– To be aged more than 18 years	– To be aged less than 18 years
	– To be involved in the caring task for at least 1 year	– To be involved in caring tasks for less than 1 year
	– >12 caring hours/week	– <12 caring hours/week
	– To have a self-assessment of stress ≥5 on a ladder from 0 to 10	– To have a self-assessment of stress <5 on a ladder from 0 to 10
Semi-formal caregivers	– To carry out care work in close contact with the family members of the older person being cared for	
	– Migrant care workers, social workers, family assistants, live-in caregivers	
	– To be aged >18 years	– To be aged <18 years
	– To be involved in the caring task for at least 1 year and for ≥12 h/week	– To be involved in caring tasks for less than 1 year and for <12 h/week
	– To be involved in the caring tasks ≥20 h/week	– To be involved in the caring tasks <20 h/week
	– To have a self-assessment of stress ≥5 on a ladder from 0 to 10	– To have a self-assessment of stress <5 on a ladder from 0 to 10
Older people with LTC needs	– People aged 65 and over	– Multi-morbid conditions must not have seriously impaired their cognition and ability to answer short questions

### 2.3 The DanceCARE intervention

The development of the intervention called “Body-mind education for stress relief”, and the use of specific body–mind techniques was defined through the results of an online survey among 140 European dance movement therapists, followed by a focus-group involving 10 experts in the field of art-based interventions and care-giving, e.g., body–mind therapists, LTC researchers and psychologists. To support the development of the program, a literature review was also conducted on the emotional needs and soft skills that caregivers of older adults should develop to cope with stress. Based on these results, seven body–mind group sessions were developed by the EADMT and WM team together, with the support of the Coordinator, which was an enriching learning exchange for both teams. The EADMT team also involved Dr. Richard Coaten, as an external advisor on DMT, dementia and LTC, to take a look at the draft training program, and give suggestions for improvement.

Specific guidelines have been developed for body–mind trainers, and translated in Italian, Greek and Spanish, containing basic instructions for the proper facilitation and management of small groups, details regarding the structure and duration of each individual workshop, and preparation of the setting. The guide then goes into detail about each session of the educational program, clearly explaining for each one what the central theme is and what needs it addresses, how the workshop unfolds, and the body–mind techniques suggested. The explanation of the sessions provides description and indications for users to understand the body work and the exercises proposed.

Sessions one, three and seven were structured more according to the WM method, and the rest were fully based on DMT approach in order to form a multi-modal progressive “body–mind” intervention. The overall set up and structure of all sessions and the therapeutic embedding followed the European DMT guidelines. The program puts into practice seven group sessions (Table 2), some of which utilize neuroscientific knowledge in calming down the body and others enhanced emotional and/or creative expression. Where deemed appropriate for the conduct of the session, possible materials and music tracks are also suggested for each session of the intervention, with links, and a description of the development of the practice and the proposed creative and body work is included. Furthermore, since this is a blended programme, for each session there is a link to a video tutorial on the DanceCARE YouTube channel to complement the in-person session. The video provides more detail on the exercises and reinforces the proposed practices and encourages you to continue moving at home.

**Table 2:** Short description of the DanceCARE intervention.

Sessions	Subject	Key take away
Session 1	Self-awareness: breathing, grounding and slowing down	Through slow breathing, you can calm down your nervous system.
Session 2	Self-confidence, body posture, mobility	Growing awareness of self on a physical and emotional level. Expanding their toolbox, with tools for selfcare. Processing information by improvising individual dance and developing their own choreography.
Session 3	Mindfulness and the surroundings (stress-management)	Being in nature, and imagining nature, releases stress
Session 4	Empathy (inwards and outwards empathy) and resilience (self-care practices)	Attunement, a base for empathy and communication.
Session 5	Emotional regulation (coping strategies)	Embodying difficult moments, give them a place to be expressed, observe them and try to deal with them through creativity.
Session 6	Communication skills, expression and listening (embodied, non-verbal and verbal communication)	We encourage elements such as trust, cooperation and openness to creativity, to achieve greater awareness of emotions and new forms of personal and social expression.
Session 7	Social interaction and creativity	You can create a connection and a pleasant, shared experience with someone, like the one you care for, through drawing, singing or dancing together.

Prior to the pilot phase, a small group of professionals—comprising two or three representatives from each country involved in the pilot project (hereinafter referred to as “mind–body trainers”)—will undergo specialized training, based on the aforementioned guidelines, on the intervention to be carried out with informal and semi-formal caregivers. All body–mind trainers have a background in DMT and are licensed through an institution recognized by the European Association of Dance Movement Therapy. All selected DMT trainers have on average over 5 years of work experience and are registered with national dance therapy associations.

Also, to ensure effective monitoring, each session will be attended by an observer—either a therapist specializing in DMT or a trainee selected at the national level through relevant associations and institutions. The observer will be provided with project guidelines, previously translated in the national language, for observing the body–mind sessions, thereby contributing to the collection of high-quality art-based and embodied material.

The intervention for the caregivers consists of 1 h and a half weekly face-to-face activities based on the DMT and WM techniques previously identified and developed in group for stimulating communication and peer networking, thereby counteracting isolation and the exacerbation of stress and other symptoms such as depression (Lauritzen et al., 2015). The frequency of the body–mind sessions has been defined during the construction phase of the programme through expert advice, but also through the collection of opinions of the caregivers themselves.

**Figure 2:**
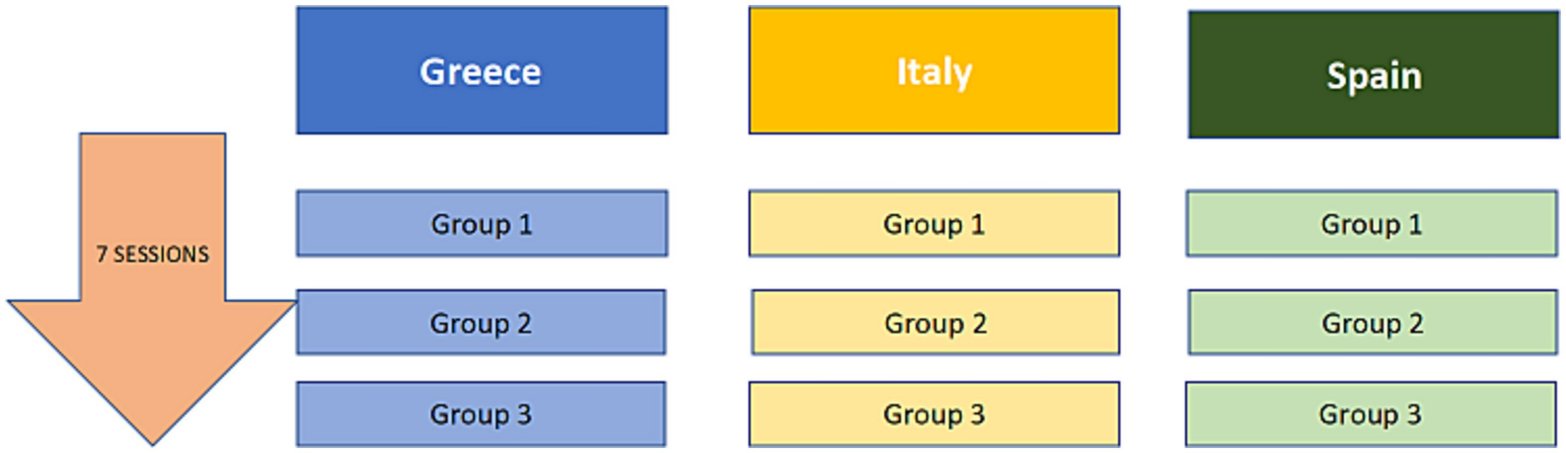
Participants’ flowchart.

As shown in Figure 2, at each of the three pilot sites, 30 informal caregivers will be divided into three groups of up to ten participants, who will attend the program simultaneously on different days and at different times of the week for approximately 3 months, taking into account holidays and other factors that could slightly affect the timeline.

The in-person activities are supplemented by online activities through nine video lessons translated into the languages of the project and based on the DanceCARE method, available on a YouTube channel^3^ and by an online platform that also includes a chat where the participants to the intervention can keep in touch between a session and another, sharing feelings and observations on their experience within the intervention.

### 2.4 Outcome variables and research tools

Employing a mixed-method design, this study will collect data on both quantitative and qualitative outcome variables, explored through proper data collection tools, at three time points (T0–T1–T2).

Table 3 provides an overview of all outcome variables, measures and research tools by target group and data collection waves. In the following two sub-paragraph quantitative and qualitative research tools are deepened.

**Table 3:** Outcomes variables and tools by target group and data collection time.

Target group	Method	Variables	Tools	Time
Informal and semi-formal caregivers of older adults Older care takers with LTC needs	Quantitative	Variables for the assessment	Common assessment tool	Screening
		Level of stress load	Zarit Burden Interview (ZBI)	T0 and T2
		Dimensions of burden	Caregiver Burden Inventory (CBI)	T0 and T2
		Depression	Beck Depression Inventory (BDI)	T0 and T2
		Satisfaction with the intervention	*Ad hoc* Likert scale-based questions	T1 and T2
	Qualitative	Personal experience with caregiving	*Ad-hoc semi-structured interview*	T0 and T2
		How and why they experience the level of stress and burden referred in the questionnaire		T0 and T2
		Perceived change of stress		T0 and T2
		*Mid-term short evaluation*: Suggestions and opinions regarding the intervention and the online learning platform and chat		T1 and T2
		Self-reflection on the body–mind experience	*Self-observation diary* for the caregivers	longitudinal
	Quantitative	Perceived quality of life	Ad hoc Likert scale-based question	T0 and T2
		Satisfaction with the care received	Yes/no and open-ended questions	T0 and T2
	Qualitative	Perceived care received	Open-ended question	T0 and T2
Body–mind trainers	Qualitative	Reporting on the body–mind sessions	*Reporting guide*	Over the intervention
		*Mid-term short evaluation*: Suggestions for improving the intervention, the online platform use and observed reactions in caregivers	Open-ended question	T1

The tools chosen in the design of this pre-post study to assess depression and psychological distress are validated versions for each country involved in the piloting. They will be sent to participants via online forms for self-administration. In addition, all interviews will be recorded and transcribed initially at the national level and then translated into English to enable a transnational analysis of the data.

For the qualitative monitoring conducted over the duration of the body–mind programme, the observers will provide data via online forms in which, in addition to noting a range of aspects of movement and dynamics in written form, they are also able to attach art-based and/or embodied materials consisting of images and/or videos relating to each individual group session. Importantly, these qualitative data will not be limited to verbal accounts; they will also include symbolic and metaphorical expressions emerging from arts-based and embodied practices, which remain grounded within the qualitative paradigm and allow for a process-oriented understanding of participants’ experiences rather than capturing them at a single point in time.

For data collection, the implementation of rigorous protocols is prioritized, and researchers are provided with detailed guidelines to minimize inter-observer variability.

A triangulation strategy will be employed using multiple data sources (e.g., tests, interviews, and observations) to validate the results.

A mid-term review will also be conducted with both participants and body–mind trainers to ensure accuracy of the study’s interpretation.

Finally, a reflexive approach is adopted to reduce researcher bias and potential prejudices in the qualitative analysis.

#### 2.4.1 Quantitative variables

Feasibility will be evaluated using predefined indicators, including recruitment rate, retention and attrition, adherence to the intervention, and completeness of data collection. Acceptability will be assessed through participant feedback. These parameters will be summarized descriptively to inform the practicality and optimization of the study design.

Concerning the assessment of the impact of the intervention on participants, the primary outcome variables are caregivers’ burden measured using, respectively, the ZBI (Zarit et al., 1987, 1980; Chattat et al., 2011) and the CBI (Novak and Guest, 1989; Valer et al., 2015). The researchers further investigate the aspect of depressive state through the BDI (Beck et al., 1961). These are self-assessment, standardized psychometric instruments, in the form of questionnaires, which can be self-completed by the subjects. It was decided to use two tests to measure caregivers’ burden because the ZBI identifies the consequences of care-giving on the caregiver’s quality of life and psychological and social condition in general, while the CBI delves deeper into the dimensions related to specific individual areas of burden, i.e., objective, psychological, physical, social, and emotional burden.

The ZBI is the most widely used instrument. Originally designed and tested in 1980, it contains 22 questions. Its brevity allows for easy administration and requires the caregiver to respond via a 5-point Likert scale from 0 (never) to 4 (almost always) according to the degree of agreement they have with the individual items. The items investigate how the patient’s disability impacts the quality of life, psychological discomfort, guilt, financial difficulties, shame and social and family difficulties of the caregiver. Consequently, the total score, which is calculated by summing the individual item response scores, is between 0 which corresponds to no care burden, to a maximum of 88 which corresponds to a maximum level of care load. Values between 21 and 40 indicate a light to moderate care burden. Values between 41 and 60 indicate a moderate to severe care burden. Values between 61 and 88 indicate a severe care burden. Scores above 24–26 identify caregivers for whom further investigation and possible support interventions would be indicated (Schreiner et al., 2006).

The CBI is a care burden assessment tool, capable of analyzing the multidimensional aspect, especially developed for caregivers of patients suffering from Alzheimer’s disease and related dementias. It is a self-report instrument, filled in by the main caregiver, i.e., the family member or caregiver who most bears the burden of caring for the patient. The caregiver is asked to answer by ticking the box that comes closest to his/her personal condition or impression. It consists of 24 items divided into five sections, i.e., objective burden, psychological burden, physical burden, social burden and emotional burden. The CBI allows different stress factors to be assessed: the time-dependent burden of care (item 1–5), which describes the burden associated with the restriction of time for the caregiver; the developmental burden (item 6–10), understood as the caregiver’s perception of feeling cut off, compared to the expectations and opportunities of their peers; the physical burden (items 11–14), which describes feelings of chronic fatigue and somatic health problems; the social burden (items 15–19), which describes the perception of role conflict; the emotional burden (items 20–24), which describes feelings towards the patient, which may be induced by unpredictable and bizarre behavior. The items are rated on a Likert scale from 0 to 4, with a minimum score of 0 and a maximum score of 96, where a higher score represents a greater burden on the caregiver. The CBI makes it possible to obtain a graphical profile of the caregiver’s burden in the various domains, to compare different subjects and to immediately observe changes in burden over time. Caregivers with the same total score may present different burden patterns. These disparate profiles address the particular social and psychological needs of caregivers and represent the different objectives of assorted intervention methods planned to relieve the specific weaknesses specified in the test. The lowest test reliability is found regarding emotional and social burden. Although no specific values have been established for the CBI, according to the Minimal Clinically Important Difference (MCID) parameters (McGlothlin and Lewis, 2014; Salas Apaza et al., 2021), a 10–20% reduction in the total score is often considered significant in similar load measures.

The results that emerge are not to be considered exhaustive for caregiver’s emotional health status, that’s why the researchers decided to do an integration of measurements concerning specifically depressive aspects through BDI.

BDI is used as a screening tool for depression and to measure behavioral manifestations and severity of depression. Validity and reliability for BDI have been tested across populations, worldwide. It can be used from ages 13 to 80 and contains 21 self-report items which individuals complete using multiple choice response formats. The BDI score ranges from 0 (no symptoms of depression) to 63 (maximum depression). Scores above 40 already indicate extreme depression. Research indicates that a 17.5% change in the BDI score from baseline indicates a clinically significant change (Button et al., 2015).

Secondary quantitative outcomes are: the degree of satisfaction of caregivers with the intervention, assessed with ad-hoc closed and open questions; the quality of life perceived by the care takers, measured with a single question that can be answered with a score from 1 to 5, where 1 is “very good” and 5 is “very bad”; the care takers’ satisfaction with the care received, measured using a yes/no question and an open-ended question to provide explanations for their answer.

Considering the quantitative instruments, the screening questionnaire collects the socio-demographic data of the caregivers and information on the type and frequency of care provided, for the purpose of inclusion or non-inclusion in the study. It also includes questions about the willingness to attend the activities offered within the intervention and the expectations the caregivers have of it, in order to be able to adapt the activities as much as possible to the needs of the participants. The screening questionnaire evaluates the older person’s abilities to understand and answer the questionnaire by means of the Lawton Instrumental Activities of Daily Living (IADL) scale (Lawton and Brody, 1969) answered by the caregiver.

The questionnaire administered to caregivers before and after the intervention (at T0 and T2) contains the ZBI, the CBI and the BDI, as described above.

#### 2.4.2 Qualitative variables

The study also explores qualitatively the caregivers’ personal experience with assistance, how and why they experience the level of stress and burden referred in the questionnaire at T0 and T2; the impact of the intervention on caregivers’ burden and older care takers’ perception of the care received; the respondents’ suggestions and opinions regarding the intervention and the online learning platform and chat. Moreover, the body–mind trainers are asked to express their impressions about the caregivers participating in the intervention and the intervention itself (e.g., method, logistics, time), at T1 and T2.

Embodied research methods involve (self) observation including movement-based tasks, somatic journaling, and sensory engagement exercises, allowing participants to express and reflect on their experiences through bodily awareness and gesture. Arts-based methods include creative outputs such as drawings and visual metaphors which serve both as data and as interpretive tools. The combination of quantitative and qualitative methods enables a more comprehensive exploration of how body–mind psycho-educational intervention may contribute to the reduction of burden. This multi-modal approach not only allows for the measurement of participants’ psychological states at a specific point in time, but it also enables the exploration of meaning beyond language, fostering a deeper understanding of participants’ lived realities through aesthetic, emotional, and corporeal dimensions.

The qualitative component invites training participants (the caregivers) and observers to describe their embodied experiences and sensations through metaphors, stories, or drawings (Aita et al., 2003). This approach, grounded in arts-based and embodied research, facilitates the expression of pre-conscious and tacit experiences (Tracy and Redden, 2015; Farquhar and Fitzpatrick, 2019), allowing implicit, non-verbal dimensions of experience to emerge (Tantia, 2021; Leavy, 2020). By moving beyond traditional methods, it supports a direct and holistic engagement with caregiving and care-receiving, enabling participants to convey sensations, emotions, and insights that might otherwise remain unspoken (Spouse, 2000; Stake and Kerr, 1994).

To this purpose, different tools were prepared to be administered over the intervention (as reported in Table 2 and detailed in Appendices 2, 3):

(a) *Ad-hoc semi-structured interview with caregivers*. The pre-intervention interview with caregivers includes open-ended questions to comment on the level of stress referred by the interviewees in the questionnaire; questions on the experience of assistance (e.g., negative and positive aspect of caregiving); stressors; available support; feelings of loneliness; awareness of signal of stress coming from the body. The post-intervention interview explores again the burden and depression scores reported by caregivers at the end of the intervention and it also asks: feelings and state of mind at the end of the journey; relationship with the body; relationship with art and expressive activities; experience with the face-to-face intervention, with the e-learning platform and the chat.
(b) *The short interview with the older care recipient*. The topic-guide includes questions asking the impressions on the care received, the emotions felt, the prevalent caregiver’s state of mind, the perceived quality of life.
(c) *Mid-term short evaluation* is requested from body–mind trainers and caregivers. The interview is administered at T1, after the fourth session of the intervention (that foresees seven face-to-face meetings) to capture any possible aspect that might decrease the efficacy, feasibility and pleasantness of the intervention and, concerning the method, the activities that work better than others.
(d) *Observation grid for the observers*. The guidelines for observers allow to capture any change in caregivers’ psycho-body awareness along the intervention sessions; how the caregivers experience enjoyment and usefulness during the intervention; how different artistic ways (movement, creative writing, drawing etc.) adopted throughout the intervention can help caregivers to express themselves and cope with the difficulties of care-giving. In the observation grid, the observers pay attention and note down various qualities of movement (flow, weight, time, space) at all stages of the session, and especially at the beginning and end. They note down their felt sense (Rappaport, 2013) during various stages of the group, and which themes emerged during the sessions. The observers are also asked to describe the whole process with metaphors, single or sequential movements, and they are asked to draw something that reflects what they take away from the session.

Other investigative and monitoring tools, while distinct from the actual study design, were developed to allow for further qualitative and experiential analysis and to facilitate reflection on the DanceCARE experience:

(e) *Reporting guide for body–mind trainers*. Across the whole duration of the body–mind part of the educational programme, trainers compile reports of every session. The body–mind trainers are asked to note down their observations on the topics that emerged, group dynamics, and highlights of the training session. The usefulness of the training guide and if they made program adaptations. They are also asked to make notations about the movements of the participants according to the Laban dimension (von Laban and Ullmann, 1971).
(f) *Self-observation diary for the caregivers* (training recipients). Caregivers are invited to keep a somatic (self-observation) diary that they can share with the trainers and the group if they wish. Prompts are given to support them in their reflection. These prompts ask about their feelings at the beginning and end of the session, as well as their interactions with their peers and the trainer and the emotional challenges that arose during the session. The caregivers are also asked if they have learned something new (like mindful breathing or communication exercises) and if and how to apply this in their care taking responsibility. Finally, the caregivers are asked which element of the session (movement, interaction, etc.) stayed with them and they would like to repeat. Then, participants are invited to express their experience of the day through a creative medium of their choices such as producing a drawing, writing a short story, or performing a movement sequence that they record on video.

### 2.5 Data management and analysis

The analysis plan for quantitative data includes a description of outcomes. The normality of continuous variable distributions will be assessed using the Shapiro–Wilk test. Depending on the results, data will be presented as either mean and standard deviation (for normally distributed variables) or median and inter-quartile range (for non-normally distributed variables). Categorical variables will be described using absolute frequencies and percentages. Comparisons between outcomes and exposures will be performed as follows: Chi-square tests for categorical variables; *t*-tests or one-way ANOVA for comparisons involving normally distributed continuous variables across groups; and non-parametric tests such as the Wilcoxon rank-sum test or Kruskal–Wallis test for non-normally distributed continuous variables. Correlations between continuous variables will be assessed using either Pearson’s or Spearman’s correlation coefficients, depending on the distribution. Although the primary aim of the study is assessing the intervention feasibility, in addition to *p*-values, effect sizes will be calculated and reported to quantify the magnitude of observed differences and associations, even if they are not statistically significant. In addition, in the absence of a control group, effect sizes for within-subject changes (pre–post) will be calculated (e.g., Cohen’s *d* for paired samples) to quantify the magnitude of change over time. These estimates will support the interpretation of the practical relevance of the findings, although causal inferences cannot be established.

Temporal comparisons (T0 vs. T2) will be conducted using paired-sample *t*-tests. If significant differences are identified in univariate analyses, multivariate models will be considered. Results will be reported as coefficients with standard errors (for continuous outcomes) or odds ratios with 95% confidence intervals (for binary outcomes), as appropriate. Model fit will be evaluated using *R*-squared or pseudo *R*-squared values.

In accordance with the intention-to-treat (ITT) principle, participants who withdraw from the study will not be replaced. Missing data in co-variates will be handled using multiple imputations via chained equations. All statistical analyses will be conducted by a statistician blinded to group allocation. A significance level of *p* < 0.05 will be used throughout. Analyses will be performed using SPSS for Windows, version 24.0 (SPSS Inc., Chicago, IL, USA).

Qualitative data are analyzed thematically following Braun and Clarke’s (2006) approach. Two independent researchers will analyze the textual data, with a third reviewer verifying the results to ensure reliability (Golafshani, 2003; Lincoln and Guba, 1985; Shenton, 2004). Observational notes, reports (and, when shared, also the diaries written by caregivers) will be compiled into narratives and then summarized across countries (Kutsche, 1998). These narratives will be coded to highlight information relevant to the research questions. The resulting codes will be grouped into overarching themes (De Munck and Sobo, 1998). To minimize subjective bias, the coding process will again be performed by two independent researchers and reviewed by a third (Golafshani, 2003).

The qualitative component plays a central role in capturing the richness and complexity of human experience through embodied and art-based research approaches. Data will be analyzed using a multi-modal interpretative framework that integrates verbal narratives with non-verbal expressions such as images, bodily movements, and metaphorical representations. These diverse forms of data will be examined through thematic analysis, supported by visual and performative analysis techniques that allow for the identification of patterns, emotional resonances, and symbolic meanings. Embodied data analysis includes attending to bodily sensations, movements and gestures, often through somatic reflection, phenomenological interpretation and metaphorical mapping (Tantia, 2020). Art-based research allows us to explore ambiguity, emotion and complexity through creative modalities; artistic outputs are analyzed through visual narrative and thematic resonance (Gupta and Zieske, 2024). To enhance methodological rigor, triangulation will be employed across data sources and modalities, systematically comparing verbal accounts with embodied and artistic expressions to identify convergence, complimentary, and divergence in meaning-making (O’Cathain et al., 2010). This process will be supported by iterative coding strategies, cross-modal thematic mapping, and the development of integrative analytic matrices that bring together textual, visual, and embodied data (Fetters et al., 2013). The embodied and artistic outputs will be treated not merely as illustrative but as epistemologically significant, offering insights that transcend linguistic articulation and contribute to a deeper understanding of participants’ lived experiences (Fraser and al Sayah, 2011).

### 2.6 Ethical considerations

#### 2.6.1 Ethical oversight and participant safeguards

This study protocol, along with the accompanying informed consent forms, has received approval from the relevant Institutional Ethical Committees in each participating country (where required). These approvals confirm that the protocol adheres to national and international standards concerning research ethics, scientific validity, and the protection of human subjects. Any substantial modifications to the study—whether pertaining to its aims, design, target population, sample size, procedures, or administrative components—that could affect participant welfare, research conduct, or study outcomes will necessitate a formal protocol amendment. Such amendments will be subject to renewed ethical review and approval by the appropriate committees prior to implementation.

#### 2.6.2 Participant information and voluntarism

Participants (i.e., informal and semi-formal caregivers and older care takers) will receive detailed information regarding the study’s aims and the structure of the dance-based intervention. This information will be conveyed through written materials (information sheets) and, when requested, through personal consultations with the study’s scientific supervisor (Dr. SS). Both formats will emphasize the voluntary nature of participation, affirming that individuals may withdraw from the study at any point without needing to provide justification and without any negative consequences. The intervention will also be clearly explained to caregivers to ensure transparency.

The nature of the intervention poses no anticipated risks to participants, nor does it involve discomfort or invasion of privacy. Intervention sessions will be facilitated by certified Dance Movement Therapy (DMT) professionals trained in the DanceCARE method. These facilitators possess extensive expertise in body–mind integration practices and are equipped to recognize and appropriately respond to any signs of participant distress. Engagement in the study will not interfere with participants’ ongoing medical treatments or supportive interventions.

#### 2.6.3 Confidentiality and data management

All data related to the study will be securely stored at the respective research sites. Participant-related documentation will be kept in locked cabinets within restricted access areas. To ensure confidentiality, all data collection instruments, case report forms, and administrative documents will use coded identification numbers rather than personal identifiers. Documents containing personal information, such as consent forms, will be stored separately from coded study data to further safeguard confidentiality. Access to digital data will be protected by password-secured systems. Any materials that could potentially link participant identities to their study data (e.g., codebooks, appointment logs) will be stored independently in locked locations with limited access.

The study will be conducted in accordance with applicable regulatory requirements and legal obligations. Study initiation will occur only after obtaining approval from the Comitato Etico Regionale delle Marche and completion of all required administrative procedures at the host institution.

All potentially eligible participants will receive comprehensive information about the study through an information sheet and informed consent form, which must be signed prior to enrollment. Participants will provide explicit consent for the processing of personal data in anonymized and aggregated form, in compliance with the General Data Protection Regulation (GDPR) and Italian Legislative Decree No. 101/2018.

Participants will be informed that study activities may be video recorded for research purposes, and specific consent for recording will be obtained. Consent for the processing of visual data will also be requested from family caregivers, when involved. Participants will also be informed that their data may be accessed by authorized personnel, members of the relevant ethics committee, and competent authorities for monitoring and verification purposes.

Written informed consent will be obtained from all participants, including consent for data storage for a period of 7 years following study completion. To ensure confidentiality, all collected data will be securely stored in a dedicated database owned by the research institute leading the study INRCA-National Institute of Health and Science on Aging. Access to the database and server will be password-protected and restricted to authorized research staff only.

The data controller is a research institute leading the study with the Principal Investigator identified as INRCA-National Institute of Health and Science on Aging. The Data Protection Officer (DPO) is a Third Party paid by the research organization.

All participant information sheets and consent forms are included in the documentation submitted for ethics approval.

The study database will be stored in electronic format and protected by password access limited to the Principal Investigator and the data manager. Paper-based questionnaires will be securely stored in locked facilities at the INRCA-National Institute of Health and Science on Aging, with access restricted to authorized personnel. All data will be retained for 7 years in compliance with GDPR requirements.

The study protocol will be submitted to the Comitato Etico Regionale delle Marche to ensure compliance with national and international ethical and legal standards, including Italian Legislative Decree such as those working in elderly care facilities No. 196/2003, the General Data Protection Regulation, the Declaration of World Medical Association (2013), and the Charter of Fundamental Rights of the European Union.

#### 2.6.4 Data integrity and dissemination

The integrity of the research requires that data collected across all pilot sites be analyzed collectively to provide a comprehensive understanding of the intervention’s outcomes. Findings derived from the study will be disseminated through peer-reviewed publications and scientific presentations. All dissemination activities will be coordinated and approved by the Steering Committee, composed of the study authors, to ensure consistency with the study’s core objectives and ethical commitments.

The qualitative components of the study—specifically, the interview guides and observation forms—are included in full to facilitate further analysis and constitute unpublished material.

Moreover, a separate article is planned to analyze the mixed results. In addition, more specifically qualitative analyses of the art-based material will be conducted at the national level by the DMT professional associations.

## 3 Discussion

### 3.1 Study novelty

The study is highly innovative because it focuses on body–mind methodologies (DMT and WM) and because it is applied in the informal care context and tries to involve a group of population which is often invisible, under-treated and difficult to involve in psycho-educational programs.

Scientific literature has demonstrated the positive effects of employing methodologies that integrates body–mind in patients with certain conditions, careers, and professionals in the social and health fields (Takahashi et al., 2023; Aithal et al., 2023; Fontanesi and Newman-Bluestein, 2024). However, there are not many studies focusing on the positive effects of DMT, and even less on its combination with the WM approach and the blended online approach, in caregivers of older people. DanceCARE project attempts to fill this research gap.

The study is also based on a truly unique survey that sought the opinions of DMT experts in Europe and had 140 respondents, which will be the subject of further publications. Using a data collection and analysis methodology enriched with the art-based method, the study is also highly qualitative and innovative, offering a very rich and three-dimensional interpretation of the results.

The research approach of this protocol therefore integrates the theory of embodied cognition, posits that emotions are not purely abstract mental states but are deeply intertwined with and influenced by our physical bodies, including their actions, postures, and sensory experiences, suggesting that perceiving and processing emotions involve a bodily re-experiencing of those emotions. This perspective challenges the traditional view of cognition as solely logical and abstract, proposing instead that thoughts and feelings are rooted in concrete experiences and interactions with the environment.

In addition to surveys and psychometric scales, this study on body–mind methodologies particular scientific rigor, the study also aims to listen to participants’ voices through the body and creative expression. Participants will engage in physical and artistic activities—movement, drawing, sensory perception, storytelling—to bring to light what is perceived, unspoken, and rich in imagery. In doing so, the design treats artistic creation and bodily awareness as legitimate sites of knowledge, rather than mere illustrations of verbal data. This kind of multi-modal, embodied inquiry responds to calls in the arts-mixed methods literature for deeper interpenetration of aesthetic, sensory, and discursive modes (Archibald and Gerber, 2018) and resonates with emerging embodied research paradigms that privilege nonverbal, felt sense, and bodily knowing.

Moreover, the effect of body–mind groups in reducing caregiver stress will be accompanied and reinforced by the possibility of viewing video tutorials on the project’s YouTube channel. Simple body–mind exercises can therefore be performed, even while seated, together with the caregiver and the older person, even at home. This blended aspect of the study, which will be evaluated simply by monitoring remote access and by the mid-term short interview, is also very innovative, especially in the field of body–mind methodologies, which, due to the very intimate and profound nature of the work involved, do not tend to include this approach.

Finally, the DanceCARE study design offers the possibility of a multi-dimensional assessment, from both a quantitative and qualitative point of view, of body–mind training to manage one’s psycho-emotional state and develop a series of soft skills in the delicate daily task of care-giving, proposing an innovative, cross-cutting, flexible, and lasting way to create a community.

### 3.2 Expected outcomes

Concerning the informal and semi-formal caregivers, it is expected that: the ZBI score will decrease by at least 10 points at T2 compared to baseline, so that it moves to the lower range of perceived burden (Schreiner et al., 2006). It is also expected that the total CBI score will decrease by 10–20% at T2 compared to baseline and the total BDI score will decrease by 17.5% compared to baseline. Moreover, it is hypothesized that the mood of caregivers will improve by at least one point.

Concerning older care takers, it is expected that the perceived quality of life will improve by at least one point; and that at least one-third of older adults will report an increase in satisfaction with the quality of care received from informal caregivers due to a better state of mind.

The qualitative component is expected to reveal how caregivers and older adults experience change through body–mind and creative practices. By engaging in interviews, embodied reflection, and arts-based tasks such as drawing, storytelling, and movement, participants are anticipated to express dimensions of care-giving that extend beyond verbal articulation. These multi-modal accounts are likely to illuminate the emotional, sensory, and relational aspects of care, highlighting shifts in body awareness, empathy, and self-connection. Such methods have been shown to access tacit and pre-linguistic knowledge (Martikainen et al., 2022), foster empowerment and voice through creative expression (Gerber et al., 2018), and provide richer, more culturally attuned understandings of lived experience (Nathan et al., 2023; Alfrey et al., 2025).

### 3.3 Study limitations, challenges and strengths

In this mixed-method study, bias can emerge from the quantitative component (e.g., randomization failures), the qualitative component (e.g., researcher subjectivity), or the integration of both. Common sources include selection bias (unrepresentative samples), measurement bias (inaccurate tools), and expectancy bias (researcher/participant preconceived notions). Given the meaning-based sampling procedure and the absence of a control group, we planned to mitigate potential biases by using various quantitative measures and combining them with qualitative data.

In particular, the proposed study presents some limitations and potential challenges that must be acknowledged. First, the recruitment process may prove demanding, as informal caregivers often face multiple constraints such as lack of time, physical exhaustion, emotional overload, transportation difficulties, and competing family responsibilities. These factors, combined with possible reluctance to participate due to privacy concerns or fear of being judged on their caregiving and/or movement abilities, may hinder enrolment. Furthermore, since recruitment largely depends on community networks and voluntary participation, there is a risk of selection bias toward caregivers who are already motivated or have more flexible schedules, which may limit the representativeness of the sample. Another challenge concerns participants’ adherence throughout the intervention. Given the vulnerability and daily burden of informal caregivers, dropouts may occur due to health deterioration, caregiving crises, or changing family circumstances.

Additionally, the small sample size and the pilot nature of the study limit the generalizability of the results. The qualitative data collection while providing deep insights may also be influenced by cultural and linguistic differences across the three participating countries, as well as by participants’ varying levels of comfort with embodied or art-based expression. Finally, although measures have been taken to standardize the protocol across pilot sites, differences in facilitation styles among DMT and WM trainers could introduce variability in intervention delivery.

Despite these limitations, this study offers valuable preliminary evidence on how body–mind psycho-educational interventions can support informal and semi-formal caregivers’ well-being and resilience, providing a foundation for larger-scale research and the development of sustainable support strategies in the long-term care context. Demonstrating the effectiveness of body–mind approaches and expressive arts Interventions in reducing stress and risk of depression in formal and informal caregivers of older people could encourage doctors to include such practices in social prescriptions (Scarpetti et al., 2024).

It would also be interesting to expand the scope of the investigation and, for example, measure certain parameters and biological markers of stress or brain functions before and after participating in the body–mind sessions of the educational program.

Certainly, having trained DMT and WM experts in the methodology, it would be desirable for the programme to be replicated, so that the associated study could reach a larger sample, thereby increasing its significance.

## Footnotes

1
https://wisemotionco.com/wisemotion-method/

3
https://www.youtube.com/@DanceCareErasmusProject
