# Supplementary material for: A mixed-method pilot study to assess the feasibility of a body–mind intervention in reducing burden and depressive symptoms of informal and semi-formal caregivers of older adults: the DanceCARE research protocol
Source: Front Psychol. 2026 May 28;17:1770820. doi: 10.3389/fpsyg.2026.1770820 (PMC13253729; doi:10.3389/fpsyg.2026.1770820)
Supplement: Supplementary file 1 [file Data_Sheet_1.PDF]

SUPPLEMENTARY MATERIALS

APPENDIX 1
(Questionnaires sent by google forms)

- **Beck's Depression Inventory (BDI)**

This depression inventory can be self-scored. The scoring scale is at the end of the questionnaire.
In the following questionnaire you will find groups of statements. Read carefully one group at a time.
group at a time. Choose from each group the statement that best describes how you felt during the past week, excluding today (Check the box to the left of the statement).
It is important that you read all statements in a group before answering.

**Beck´s Depression Inventory (BDI)**	
**1. Sadness**	
0. I do not feel sad.	
1. I feel sad.	
2. I am sad all the time and I can't snap out of it.	
3. I am so sad and unhappy that I can't stand it.	
**2. Pessimism**	
0. I am not particularly discouraged about the future.	
1. I feel discouraged about the future.	
2. I feel I have nothing to look forward to.	
3. I feel the future is hopeless and that things cannot improve.	
**3. Failure**	
0. I do not feel like a failure.	
1. I feel I have failed more than the average person.	
2. As I look back on my life, all I can see is a lot of failures.	
3. I feel I am a complete failure as a person.	
**4. Loss of pleasure**	
0. I get as much satisfaction out of things as I used to.	
1. I don't enjoy things the way I used to.	
2. I don't get real satisfaction out of anything anymore.	
3. I am dissatisfied or bored with everything.	
**5. Feelings of guilt**	
0. I don't feel particularly guilty.	
1. I feel guilty a good part of the time.	
2. I feel quite guilty most of the time.	
3. I feel guilty all of the time.	
**6. Feelings of punishment**	
0. I don't feel I am being punished.	
1. I feel I may be punished.	
2. I expect to be punished.	
3. I feel I am being punished.	
**7. Self-dissatisfaction**	
0. I don't feel disappointed in myself.	
1. I am disappointed in myself.	
2. I am disgusted with myself.	
3. I hate myself.	
**8. Self-criticism**	
0. I don't feel I am any worse than anybody else.	

1. I am critical of myself for my weaknesses or mistakes.	
2. I blame myself all the time for my faults.	
3. I blame myself for everything bad that happens.	
**9. Suicidal thoughts or desires**	
0. I don’t have any thoughts of killing myself.	
1. I have thoughts of killing myself, but I would not carry them out.	
2. I would like to kill myself.	
3. I would kill myself if I had the chance.	
**10. Crying**	
0. I don’t cry any more than usual.	
1. I cry more now than I used to.	
2. I cry all the time now.	
3. I used to be able to cry, but now I can’t even cry though I want to.	
**11. Concern**	
0. I am no more irritated by things than I ever was.	
1. I am slightly more irritated now than usual.	
2. I am quite annoyed or irritated a good deal of the time.	
3. I feel irritated all the time.	
**12. Loss of interest**	
0. I have not lost interest in other people.	
1. I am less interested in other people than I used to be.	
2. I have lost most of my interest in other people.	
3. I have lost all of my interest in other people.	
**13. Indecision**	
0. I make decisions about as well as I ever could.	
1. I put off making decisions more than I used to.	
2. I have greater difficulty in making decisions more than I used to.	
3. I can’t make decisions at all anymore.	
**14. Devaluation**	
0. I don’t feel that I look any worse than I used to.	
1. I am worried that I am looking old or unattractive.	
2. I feel there are permanent changes in my appearance that make me look unattractive.	
3. I believe that I look ugly.	
**15. Energy loss**	
0. I can work about as well as before.	
1. It takes an extra effort to get started at doing something.	
2. I have to push myself very hard to do anything.	
3. I can’t do any work at all.	
**16. Changes in sleep habits**	
0. I can sleep as well as usual.	
1. I don’t sleep as well as I used to.	
2. I wake up 1-2 hours earlier than usual and find it hard to get back to sleep.	
3. I wake up several hours earlier than I used to and cannot get back to sleep.	
**17. Tiredness or fatigue**	
0. I don’t get more tired than usual.	
1. I get tires more easily than I used to.	
2. I get tired from soing almost anything.	
3. I am too tired to do anything.	

**18. Changes in appetite**	
0. My appetite is no worse than usual.	
1. My appetite is not as good as it used to be.	
2. My appetite is much worse now.	
3. I have no appetite at all anymore.	
**19. Weight**	
0. I haven't lost much weight, if any, lately.	
1. I have lost more than fice pounds.	
2. I have lost more than ten pounds.	
3. I have lost more than fifteen pounds.	
**20. Health**	
0. I am no more worried about my health than usual.	
1. I am worried about physical problems like aches, pains, upset stomach, or constipation.	
2. I am very worried about physical problems and it's hard to think of much else.	
3. I am so worried about my physical problems that I cannot think of anything else.	
**21. Loss of interest in sex**	
0. I have not noticed any recent change in my interest in sex.	
1. I am less interested in sex than I used to be.	
2. I have almost no interest in sex.	
3. I have lost interest in sex completely.	

- **Zarit Caregiver Burden Assessment (Revised, 22-items)**

The following is a list of statements that reflect the way people sometimes feel when caring for another person.
After reading each statement, indicate how often you experience the listed feelings by ticking the box that best corresponds to the frequency of these feelings.

0- Never	1- Rarely	2- Sometimes	3 - Frequently	4 -Nearly Always

**Zarit Caregiver Burden Assessment**					
	0	1	2	3	4
1. Do you feel stressed between caring for your relative and trying to meet other responsibilities for your family or work?					
2. Do you feel embarrassed about your relative's behavior?					
3. Do you feel angry when you are around your relative?					
4. Do you feel that your relative currently affects your relationship with other family members or friends in a negative way?					
5. Are you afraid of what the future holds for your relative?					
6. Do you feel strained when you are around your relative?					
7. Do you feel that you do not have as much privacy as you would like because of your relative?					
8. Do you feel that your social life has suffered because you are caring for your relative?					
9. Do you feel uncomfortable about having friends over because of your relative?					

10. Do you feel that you have lost control of your life since your relative's illness?					
11. Do you wish you could just leave the care of your relative to someone else?					
12. Do you feel uncertain about what to do about your relative?					
13. Do you feel that you should be doing more for your relative?					
14. Do you feel you could do a better job in caring for your relative?					
15. Overall, how burdened do you feel in caring for your relative?					
16. Do you feel that your relative asks for more help than (s)he needs?					
17. Do you feel that because of the time you spend with your relative that you do not have enough time for yourself?					
18. Do you feel your relative is dependent upon you?					
19. Do you feel your health has suffered because of your involvement with your relative?					
20. Do you feel that your relative seems to expect you to take care of him/her as if you were the only one he/she could depend on?					
21. Do you feel that you will be unable to take care of your relative much longer?					
22. Do you feel that you do not have enough money to care for your relative in addition to the rest of your expenses?					

- **Caregiver Burden Inventory**

We invite you to read each statement carefully. After thinking about the content of each one, please, indicate how often you experience the feeling or the situation described. To do so, tick on the box that corresponds better to your experience by using the following scale.

0 - Not at all descriptive	1	2	3	4- Very descriptive

Caregiver Burden Inventory					
**Factor 1: Time-Dependence Burden**	0	1	2	3	4
1. My care receiver needs my help to perform many daily tasks.					
2. My care receiver is dependent on me.					
3. I have to watch my care receiver constantly.					
4. I have to help my care receiver with many basic functions.					
5. I don't have a minute's break from my caregiving chores.					
**Factor 2: Developmental Burden**	0	1	2	3	4
1. I feel that I am missing out on life.					
2. I wish I could escape from this situation.					
3. My social life has suffered.					
4. I feel emotionally drained due to caring for my care receiver.					

5. I expected that things would be a different at this point in my life.					
**Factor 3: Physical Burden**	0	1	2	3	4
1. I'm not getting enough sleep.					
2. My health has suffered					
3. Caregiving has made me physically sick.					
4. I'm physically tired.					
**Factor 4: Social Burden**	0	1	2	3	4
1. I don't get along as well as I used to with other members of my family.					
2. My efforts as a caregiver are not appreciated by other members of my family.					
3. I've had problems with my marriage.					
4. I don't do my tasks at work as well as I used to.					
5. I feel resentful towards other family members who could help, but don't.					
**Factor 5: Emotional Burden**	0	1	2	3	4
1. I am ashamed of the behavior of the person I care for.					
2. I feel ashamed of the person I take care of.					
3. I resent the person I care for.					
4. I feel uncomfortable when I have friends visiting.					
5. My interactions with the person I care for make me angry.					

APPENDIX 2

A) Pre-Post interview with participant caregivers (T0 and T2)

1.	You are caring for……………………………….. [INSERT RELATIONSHIP WITH THE CARED FOR PERSONA E.G. YOUR FATHER OR, IN CASE OF A MIGRANT CARE WORKER, A MAN, WITH E.G. ALZHEIMER DISEASE].
2.	In the case of an INFORMAL CAREGIVER i.e. a FAMILY CAREGIVER, what are the reasons for caring? In the case of a SEMI-FORMAL CAREGIVER i.e. a (migrant) care worker: What motivates you to do this work?
3.	Based on the answers you gave to the questionnaire, I realised that you reported a (low/high/medium) level of burden (ZARIT). What do you think are the most important reasons for this outcome?
4.	I noticed that your mood BDI (Beck Depression inventory) is [..........................] that means that [..]. How do you comment on these outcomes? Are

	you willing to share a bit on this topic?
5.	I would like to comment with you on the results of the Caregiver Burden Inventory (CBI) you filled in. I noticed that [the interviewer comment the results on the dimensions (time, psychological, physical, emotional and social) if the CBI and ask the interviewee his/her own explanation of the response].
6.	Are there positive aspects in caregiving? Which are they? What do you enjoy about caregiving?
7.	Which are the more stressful factors in caregiving (e.g. activities, relationship, pain…)?
8.	Which supports can you count on in your role of carer? Which people can you count on? Which services/support/benefit (public and/or private)?
9.	Do you ever feel isolated or lonely?
10.	Do you have time, set apart, just for yourself? And if so, what do you prefer to do in this time to feel better? Which type of activities do you do for taking care of yourself, having respite, regenerating yourself? [If the person finds it difficult to speak and tell or to focus on the answer you can ask: Do you practice any physical activity such as running, gym, walking, hiking, dancing?].

5.	
11.	How aware do you think you are of your body and the signals coming from your body? From 0 (very little) to 5 (very much). Can you specify which signals you pick up (for example: if my body is cold or when i am stressed i get pain in my neck, or sometimes I notice I hold my breath)?

Welcome back to the end of the DanceCARE project. We are curious to know how this experience went for you.

1.	How do you feel at the end of the journey?
2.	Has anything changed at the level of your psycho-body awareness? If so, what? How did the change occur?
3.	With regard to the 7 body-mind training sessions, what has been most helpful for you?
4.	How do you now view your relationship with creativity and art?
5.	What do you do in your spare time to feel better? Have your activities changed compared

	to before start of the project? If yes, in what way?
6.	PLATFORM: What do you think of the platform with the videos? Were they useful? Will you also use them in the future?
7.	CHAT: How useful was chat for exchanging information? Do you plan to continue using it in the future?
8.	Did you find the activation of self-help groups useful? If yes, do you plan to continue participating or otherwise stay in contact with other carers?

Now I invite you to comment on the results of the questionnaires and the differences between the beginning and end of the project.

1.	Based on the answers you gave to the post-questionnaire, I realised that you reported a [lower/higher] level of burden (ZARIT) compared with the pre-test. What do you think are the most important reasons for this outcome?
2.	I noticed that your mood post-test BDI (Beck Depression Inventory) is [.........................] that means that [.....................................] How do you comment on these outcomes and the changes compared to pre-test? Are you willing to share a bit on this topic?

3.	I finally would like to comment with you on the results of the <u>Caregiver Burden Inventory</u> (<u>CBI</u>) you filled in. I noticed that [the interviewer comments the resultss on the dimensions pre and post (time, psychological, physical, emotional and social) of the CBI and asks the interviewee his/her explanation of the response.

B) **Mid-term short evaluation at T1**

<u>For caregivers/beneficiaries</u>

We are in the middle of the DanceCARE intervention and I would like to know how you are and your impressions about this experience. To do so, I will ask you a few questions. If you agree, our conversation will be recorded so that I can pay you all my attention now and listen to the interview later. So, we can start….

1	What are you loving most about the DanceCARE programme? And why?
2	What are you least liking about the DanceCARE programme? And why?
3	How did you feel till now during the intervention sessions?
4	Would you like to give us some suggestions for improvement? Is there anything that you would like to change in contents, methods, logistics (e.g. times and location)?
5	Do you think the online platform and videos helped? If yes - if no, why?
6	Are you using the project chat to communicate?
7	If yes, how useful do you find it on a scale of 1 to 5 (1: not at all, 5: very much)

For body-mind trainers

We are in the middle of the DanceCARE intervention and I would like to know your impressions about the training and the beneficiaries. To do so, I will ask you a few questions. If you agree, our conversation will be recorded so that I can pay you all my attention now and listen to the interview later. So, we can start….

1	Which reactions did you observe in the caregivers who are participating in the session?
2	Which is the activity that worked better?
3	How did you feel during the sessions?
4	Would you like to give us some suggestions for improvement? Is there anything that you would like to change in contents, methods, logistics (e.g. times and location)?
5	Do you think the online platform and videos helped? If yes - if no, why?
6	Are you using the chat to communicate?
7	If yes, how useful do you find it on a scale of 1 to 5 (1: not at all, 5: very much)

APPENDIX 3

GUIDED OBSERVATION OF MOVEMENTS AND EMOTIONS (for trainees/observers)
GENERAL INSTRUCTIONS
Preparing to observe:
Before starting the observation session it is necessary to be in a state of awareness (embodiment), which consists of centering, with a regular and deep breath, and good rooting. Being in the here and now allows the observer to listen, to have a space inside himself to welcome the other, the gestures, the movements and the state of the group.

Below you find a guided observation grid. We understand it is not possible to pay attention to all the aspects of the different phases. That is ok, we are not expecting you to. Use your intuition, and see what draws your attention. The grid is developed to support you as an observer, in finding structure in your observations.
We suggest you pay attention to group rhythm, flow, qualities, and use of space especially at the beginning and end of each session.

Under the observation grid, you find other open questions, and artistic assignments to capture your observation in a more embodied way, through a drawing, song, a story etc.
SHORT EXPLANATION OF GRID TERMS

SPACE:

Planes of space (vertical, horizontal, sagittal)
Levels (high, medium, low)
Kinesphere (proximal, medium, wide)
The use of TIME can be PROLONGED or URGENT. It connects to the sagittal plane of space and is the factor that expresses intuition and decision.
FLOW can be FREE or TENSE/HOLDED. It is the emotional and most important factor behind movement and expression
EFFORT: How does the group move? What is the intention and the expressive manifestation of the movement? WEIGHT can be STRONG or LIGHT. It connects to the plane of vertical space and is the factor that expresses the intention of the movement.
In real life these factors do not appear separately, but are in combination with each other and give rise to all expressive and functional movements.

**ANNEX 3 - OBSERVATION GRID (for observers) - DanceCARE**		
Date of the session		
FULL NAME OF THE OBSERVER		
PHASES OF THE WORKSHOP	What and how to observe?	
1- check-in	A) Use of the SPACE (personal and general)	
	B. TIME and rhythm of the group	
	C. How is the FLOW?	
	D. EFFORT/QUALITY	
	E. How do I feel while the group is moving? Can I write down 1 feeling/word for this phase?	
2- warming-up	A) Use of the SPACE (personal and general)	
	B. TIME and rhythm of the group	
	C. How is the FLOW?	
	D. EFFORT/QUALITY	
	E. How do I feel while the group is moving? Can I write down 1 feeling/word for this phase?	
3- process	A) Use of the SPACE (personal and general)	
	B. TIME and rhythm of the group	
	C. How is the FLOW?	
	D. EFFORT/QUALITY	
	E. How do I feel while the group is moving? Can I write down 1 feeling/word for this phase?	
4- closure	A) Use of the SPACE (personal and general)	

	B. TIME and rhythm of the group	
	C. How is the FLOW?	
	EFFORT/QUALITY	
	How do I feel while the group is moving? Can I write down 1 feeling/word for this phase?	
5- Check out	A) Use of the SPACE (personal and general)	
	D. TIME and rhythm of the group	
	E. How is the FLOW?	
	EFFORT/QUALITY	
	How do I feel while the group is moving? Can I write down 1 feeling/word for this phase?	

<u>SUGGESTED QUESTIONS FOR OBSERVERS</u>
<u>(to be completed immediately after the end of each session)</u>

1.	What is your general impression(s) of the session?
2.	Which is/are the important theme(s) that emerged during the process?
3.	Was there a moment during the session that touched you and you felt had a deep impact on a participant (or several participants)?

<u>Art based assignment after the observation</u>

4.	Can you describe the whole process of the session in metaphor or a movement (sequence9? If you choose for the movement (sequence) we ask you to record yourself?

5.	Please, take some minutes to draw what you take away from this session.
