# Supplementary material for: A mixed-method pilot study to assess the feasibility of a body–mind intervention in reducing burden and depressive symptoms of informal and semi-formal caregivers of older adults: the DanceCARE research protocol
Source: Front Psychol. 2026 May 28;17:1770820. doi: 10.3389/fpsyg.2026.1770820 (PMC13253729; doi:10.3389/fpsyg.2026.1770820)
Supplement: Supplementary file 2 [file Data_Sheet_2.PDF]

SUPPLEMENTARY MATERIALS - PART 2 tables

TABLE 1 Participants' selection criteria

Type of participants	Inclusion criteria	Exclusion criteria
Informal caregivers	- to provide care for an older family member by choice and/or necessity, without any kind of remuneration	- to get a pay for caregiving
	- to be aged more than 18 years	- to be aged less than 18 years
	- to be involved in the caring task for at least one year	- to be involved in caring tasks for less than one year
	- to be involved in the caring tasks at least 12 hours/week	- to be involved in the caring tasks for less than 12 hours/week
	- to have a self-assessment of stress= or > than 5 on a ladder from 0 to 10	- to have a self-assessment of stress < than 5 on a ladder from 0 to 10
Semi-formal caregivers	- to carry out care work in close contact with the family members of the older person being cared for	
	- migrant care workers, social workers, family assistant, live-in caregivers	
	- to be more than 18 years old	- to be less than 18 years old
	- to be involved in the caring task for at least 1 year	- to be involved in the caring tasks for less than 12 hours/week- to be involved in caring tasks for less than one year
	- to be involved in the caring tasks at least 20 hours/week	- to be involved in the caring tasks less than 20 hours/week
	to have a self-assessment of stress= or > than 5 on a ladder from 0 to 10	to have a self-assessment of stress < than 5 on a ladder from 0 to 10
Older people with LTC needs	- people aged 65 and over	- multi-morbid conditions must not have seriously impaired their cognition and ability to answer short questions

TABLE 2 Short description of the DanceCARE intervention

Sessions	Subject
Session 1	Self-awareness: Breathing, grounding and slowing down
Session 2	Self-confidence body posture, mobility
Session 3	Mindfulness and the surroundings (stress-management)
Session 4	Empathy (inwards and outwards empathy) and resilience (self-care

	practices)
Session 5	Emotional regulation (coping strategies)
Session 6	Communication skills, expression and listening (embodied, non-verbal and verbal communication)
Session 7	Social interaction and creativity

TABLE 3 Outcomes variables and tools by target group and data collection time

Target group	Method	Variables	Tools	Time
Informal and semi-formal caregivers of older adults	Quantitative	Variables for the assessment of the inclusion/exclusion criteria	Common assessment tool	Screening
		Level of stress load	**ZBI**	T0 and T2
		Dimensions of burden	**CBI**	T0 and T2
		Depression	**BDI**	T0 and T2
		Satisfaction with the intervention	Ad hoc Likert scale-based questions	T1 and T2
	Qualitative	Personal experience with caregiving	**Ad-hoc semi-structured interview**	T0 and T2
		How and why they experience the level of stress and burden referred in the questionnaire		T0 and T2
		Perceived change of stress		T0 and T2
		**Mid-term short evaluation:** Suggestions and opinions regarding the intervention and the online learning platform and chat		T1 and T2

		Self-reflection on the body-mind experience	**Self-observation diary** for the caregivers	Over the intervention
Older care takers with LTC needs	Quantitative	Perceived quality of life	Ad hoc Likert scale-based question	T0 and T2
		Satisfaction with the care received	Yes/no and open-ended questions	T0 and T2
	Qualitative	Perceived care received	Open-ended question	T0 and T2
Body-mind trainers	Qualitative	Reporting on the body-mind sessions	**Reporting guide**	Over the intervention
		**Mid-term short evaluation:** Suggestions for improving the intervention and observed reactions in caregivers	Open-ended question	T1
